# Risk Factors for Early Poor Outcomes in In-hospital Intracranial Hemorrhage: A Retrospective Cohort Study

**DOI:** 10.1007/s12028-025-02306-0

**Published:** 2025-07-01

**Authors:** Tian Qu, Shengde Li, Xiang Zhou, Qi Miao, Jun Ni, Bin Peng

**Affiliations:** 1 Department of Neurology, State Key Laboratory of Complex Severe and Rare Diseases, Peking Union Medical College Hospital, Chinese Academy of Medical Science and Peking Union Medical College, Beijing, China; 2 Department of Information Center, State Key Laboratory of Complex Severe and Rare Diseases, Peking Union Medical College Hospital, Chinese Academy of Medical Science and Peking Union Medical College, Beijing, China; 3 Department of Cardiac Surgery, State Key Laboratory of Complex Severe and Rare Diseases, Peking Union Medical College Hospital, Chinese Academy of Medical Science and Peking Union Medical College, Beijing, China

**Keywords:** Intracranial hemorrhage, In-hospital stroke, Prognosis, Risk factors

## Abstract

**Background:**

Compared to in-hospital ischemic stroke, the prognosis of in-hospital intracranial hemorrhage (IH-ICH) remains poorly understood. We aimed to analyze the risk factors for early poor outcomes and propose a novel predictive nomogram for in-hospital ICH.

**Methods:**

We retrospectively analyzed data of patients with in-hospital ICH treated in our hospital between 2014 and 2022. Baseline demographics, comorbidities, clinical characteristics, and outcomes were collected. The early poor outcome was defined as in-hospital death or discharge against medical advice. Univariate and multivariate logistic regressions were used to identify the risk factors and then construct a nomogram. The nomogram was compared with the ICH score in terms of predictive ability.

**Results:**

A total of 169 patients were included; the median age was 57.0 (interquartile range 40.0–67.0) years, and 84 (49.7%) patients were male. Among the cohort, 135 patients had intraparenchymal hemorrhage, 27 had subarachnoid hemorrhage, 1 had intraventricular hemorrhage, 5 had subdural hemorrhage, and 1 had epidural hemorrhage. Overall, 96 (56.8%) patients developed an early poor outcome. Multivariate logistic regression identified prior spontaneous extracranial hemorrhage (ECH), baseline modified Rankin Scale (mRS) score ≥ 4, baseline Glasgow Coma Scale (GCS) score ≤ 8, and systemic disease etiology as independent risk factors for early poor outcomes. The IH-ICH nomogram, developed based on these risk factors, had good calibration and superior predictive performance compared to the conventional ICH score (area under the receiver operating characteristic curve 0.894 vs. 0.743, *p* < 0.001). Besides, the decision curve analysis curves revealed greater positive net benefit of the model than the ICH score.

**Conclusions:**

Patients with prior ECH, severe coma (GCS score ≤ 8), poor functional status (mRS score ≥ 4), and systemic disease etiology face a significant risk of early poor outcomes. The IH-ICH nomogram incorporating these factors offers a promising tool for identifying high-risk patients with in-hospital ICH, thereby contributing to improved patient care and resource allocation in neurology and critical care settings.

**Supplementary Information:**

The online version contains supplementary material available at https://doi.org/10.1007/s12028-025-02306-0.

## Introduction

Intracranial hemorrhage (ICH) is a life-threatening and debilitating form of cerebrovascular disease, affecting approximately 4.5 million people globally each year [1]. Despite advances in diagnostic imaging and critical care management, treatment options for ICH remain limited, and outcomes are often poor, with high mortality and reduced quality of life [2, 3]. In-hospital ICH refers to spontaneous, nontraumatic ICH occurring during hospitalization, often in patients receiving anticoagulant therapy, following cardiac surgery, or in patients with serious underlying comorbidities [4–6]. Compared to stroke occurring in the community, the prognosis of in-hospital stroke is significantly worse, with double the 30-day mortality, greater disability, and a diminished quality of life among survivors [6–8]. Although the incidence of in-hospital ICH is relatively low, its fatality rate far exceeds that of in-hospital ischemic strokes [9, 10]. In recent years, growing attention has been paid to in-hospital ischemic stroke [11–13]; however, studies focusing on in-hospital ICH remain scarce. Therefore, further research is urgently needed to comprehensively investigate the etiology, mechanisms, and prognosis of in-hospital ICH.

In this study, we retrospectively analyzed a cohort of patients with in-hospital ICH treated at our institution between 2014 and 2022 to investigate the risk factors for early poor outcomes and construct a novel predictive nomogram for in-hospital ICH. Our aim was to improve the early identification and screening of critical patients with in-hospital ICH and provide a more reliable basis for clinical decision- making.

## Methods

### Study population

We retrospectively reviewed patients consecutively admitted to Peking Union Medical College Hospital who developed spontaneous ICH during hospitalization for another non-ICH cause between November 1, 2014, and October 31, 2022. Inclusion criteria were as follows: (1) age ≥ 18 years; (2) spontaneous ICH development during hospitalization for other non-ICH diseases; and (3) ICH confirmed by computed tomography/magnetic resonance imaging, including hemorrhagic transformation of ischemic stroke (HT-IS) or intracerebral, ventricular, subdural, epidural, or subarachnoid hemorrhage. Exclusion criteria were as follows: (1) traumatic ICH, (2) ICH related to intracranial surgery, and (3) pure cerebral microbleeds on susceptibility-weighted imaging.

### Measures

Baseline data, including demographics, comorbidities (e.g., hypertension, diabetes mellitus, dyslipidemia, heart disease, and prior stroke), a history of spontaneous extracranial hemorrhage (ECH) within 4 weeks, and the prior use of antiplatelet and anticoagulant agents, were collected. Laboratory parameters were retrieved from medical records: hemoglobin (HGB), platelet count (PLT), white blood cell count (WBC), alanine aminotransferase, serum creatinine (SCr), international normalized ratio (INR), activated partial thromboplastin time, and thrombin time. Anemia was defined as HGB < 120 g/L in women and < 130 g/L in men. Thrombocytopenia was defined as PLT < 100 × 10^9^/L. Scores such as the modified Rankin Scale (mRS), Glasgow Coma Scale (GCS), and ICH score were collected at the time of ICH onset according to standard guidelines [14–16], serving as baseline indicators to assess the patients’ condition. The etiology of ICH was assessed based on clinical history, neuroimaging findings, laboratory results, and review of medical records. We made slight modifications to the SMASH-U classification [17] when categorizing ICH etiology. The definitions of several key etiologies are as follows: (1) structural vascular lesions: hemorrhages due to arteriovenous malformations, dural arteriovenous fistulas, cerebral aneurysms, or cavernous malformations confirmed by neurovascular imaging or surgical/pathological evidence; (2) medication related: hemorrhages occurring in patients with recent use of anticoagulant or antiplatelet agents without evidence of structural or systemic disease; (3) systemic disease related: hemorrhages secondary to systemic conditions such as chronic liver disease, hematologic malignancies, uremia, vasculitis, or sepsis-associated coagulopathy; (4) multiple causes: when more than one plausible etiology was identified (e.g., a patient on anticoagulation who also had liver dysfunction contributing to coagulopathy), the case was classified as having multiple causes; and (5) undetermined: cases in which no clear etiology could be established after complete review of clinical, laboratory, and imaging data and without relevant medication exposure. Hemorrhage location, volume (calculated as [a × b × c]/2, in mL), and the number of lesions were documented. Follow-up data, including GCS and mRS scores, complications, and clinical events, were collected until discharge. All assessments were confirmed by an independent senior neurologist panel.

### Outcomes

The primary early poor outcome was defined as death from any cause during hospitalization or discharge against medical advice (DAMA). Patients transferred to another hospital or rehabilitation center were not classified as DAMA. We used the composite outcome of in-hospital death or DAMA because it is common in China for patients to withdraw from treatment at an unfavorable or terminal stage due to a cultural reluctance to pass away in hospitals [18]. Based on this definition, patients were categorized into two groups: the early poor outcome group and the early good outcome group. An independent committee led by Dr. Jun Ni confirmed all outcomes.

### Statistical Analysis

Categorical variables were displayed as n (%) and were compared using the χ^2^ test or Fisher’s exact test as appropriate. Continuous variables are expressed as mean ± standard deviation or as median (interquartile range [IQR]) based on their distribution, as evaluated using the Shapiro–Wilk test. Student’s *t*‐test or the Mann–Whitney *U*-test was used to compare the continuous variables. Continuous variables were transformed into categorical variables primarily according to clinical experience (e.g., GCS scores were divided into two groups based on the presence of severe coma: ≤ 8 and > 8). Variables with *p* values < 0.05 in the univariate logistic regression analysis were included in the multivariate logistic regression model to identify the independent risk factors for early poor outcomes in patients with in-hospital ICH. We used a forward elimination procedure in the multivariate logistic regression analysis to arrive at a minimal model including only variables with *p* values < 0.10. Potential multicollinearity was evaluated using variance inflation factor (VIF), and a VIF ≤ 3 was considered indicative of noncollinearity. Hematoma volume is believed to be associated with the prognosis of ICH [19]. Given the relatively high percentage of incalculable hematoma volume data and the small sample size of patients with dyslipidemia, we excluded the variables of hematoma volume and dyslipidemia in the multivariate logistic regression models. Sensitivity analyses by constructing additional models that included baseline hematoma volume and dyslipidemia were conducted to assess the robustness. A subgroup analysis examined whether these independent risk factors for poor prognosis varied by age (≤ 45 vs. ≥ 46 years), sex, baseline hematoma volume, presence of intraventricular hemorrhage (IVH), thrombocytopenia, intensive care unit (ICU) admission, and pneumonia.

A nomogram named the IH-ICH nomogram was formulated based on all independent risk factors that predicted poor outcomes in the final model. The discriminative ability of the IH-ICH nomogram was evaluated by calculating the area under the receiver operating characteristic curve (AUC-ROC). A comparison of AUC-ROC values was performed to assess the predictive power of the IH-ICH nomogram relative to the ICH score. Calibration was assessed through a calibration plot generated with 1,000 bootstrap resamples. Additionally, a decision curve analysis (DCA) was conducted to evaluate the clinical utility of the IH-ICH nomogram and the ICH score by quantifying the net benefit across a range of threshold probabilities within this cohort. Furthermore, subgroup analyses were performed to further assess the robustness of the model.

Statistical analyses were performed using SAS version 9.3 (SAS Institute, Inc.) and R software version 4.4.1. The nomogram and calibration plot were constructed using the “rms” package; and the DCA was established using the “rmda” package. A two-tailed value of *P* < 0.05 was considered as statistically significant in this study.

## Results

### Study Population Characteristics

**Fig. 1:**
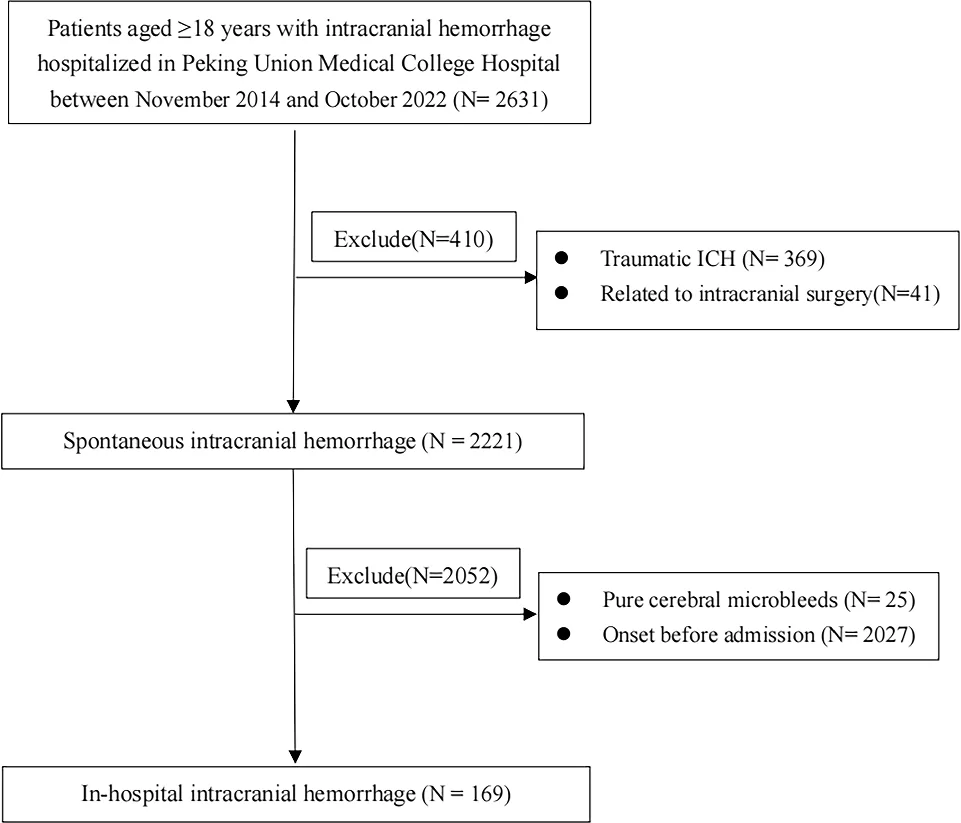
Flowchart of in-hospital intracranial hemorrhage (ICH) cohort in Peking Union Medical College Hospital

As shown in Fig. 1, a total of 169 patients were included in final analysis. Forty-six (27.2%) patients died from any causes during hospitalization, with the causes of death shown in eFigure 1. Fifty (29.6%) patients were discharged against medical advice, and 73 (43.2%) patients were discharged or transferred in a stable condition. Thus, 96 (56.8%) patients in the cohort developed an early poor outcome. The median age at ICH onset was 57.0 (IQR 40.0–67.0) years, and 84 (49.7%) patients were male. Basic demographic and medical history data are displayed in Table 1. Patients with an early poor outcome were younger (53.5 [IQR 37–62.5] years vs. 60 [IQR 48–72] years; *p* = 0.002) and had a relatively lower prevalence of dyslipidemia (4.2% vs. 16.4%; *p* = 0.007), prior ischemic stroke (25.0% vs. 48.0%; *p* = 0.002), and antiplatelet drug use within 2 weeks (14.6% vs. 38.4%; *p* < 0.001). Conversely, they had a higher prevalence of chronic renal failure (11.5% vs. 2.7%; *p* = 0.035), hematologic tumors (22.9% vs. 6.9%; *p* = 0.005), and prior ECH (45.8% vs. 12.3%; *p* < 0.001).

**Table 1:** Demographic, and medical history of patients with in-hospital ICH

	Overall N = 169	Group EGO N = 73	Group EPO N = 96	P value
Age, years	57(40–67)	60(48–72)	53.5(37,62.5)	**0.002**
Age ≤ 45 years	52(30.77)	15(20.55)	37(38.54)	**0.012**
Sex, male	84(49.70)	33(45.21)	51(53.13)	0.308
BMI	23.34 ± 3.61	23.32 ± 4.01	23.36 ± 3.31	0.946
Overweight^a^	60(35.50)	26(35.62)	34(35.42)	0.979
Hypertension	81(47.93)	41(56.16)	40(41.67)	0.062
Diabetes mellitus	33(19.53)	15(20.55)	18(18.75)	0.770
Dyslipidemia	16(9.47)	12(16.44)	4(4.17)	**0.007**
Prior stroke	64(37.87)	39(53.42)	25(26.04)	**0.003**
Ischemic stroke	59(34.91)	35(47.95)	24(25.00)	**0.002**
Hemorrhagic stroke	2(1.18)	2(2.74)	0	0.185
History of heart disease	61(36.09)	29(39.73)	32(33.33)	0.391
CHD	25(14.79)	11(15.07)	14(14.58)	0.930
AF	16(9.47)	9(12.33)	7(7.29)	0.268
IE	11(6.51)	5(6.85)	6(6.25)	0.876
VD	8(4.73)	5(6.85)	3(3.13)	0.259
History of Autoimmune disease	37(21.89)	17(23.29)	20(20.83)	0.702
SLE	18(10.65)	6(8.22)	12(12.50)	0.372
Vasculitis	9(5.33)	5(6.85)	4(4.17)	0.442
APS	5(2.96)	3(4.11)	2(2.08)	0.441
Chronic renal failure	13(7.69)	2(2.74)	11(11.46)	**0.035**
Renal dialysis	6(3.55)	2(2.74)	4(4.17)	0.700
Malignant tumor	46(27.22)	12(16.44)	34(35.42)	**0.006**
Hematologic tumor	27(15.98)	5(6.85)	22(22.92)	**0.005**
Smoking, ever	48(28.40)	16(21.92)	32(33.33)	0.103
Regular drinking, ever	24(14.20)	10(13.70)	14(14.58)	0.870
Family history of cardio-vascular disease^b^	15(8.88)	9(12.33)	6(6.25)	0.169
Prior ECH^c^	53(31.36)	9(12.33)	44(45.83)	** < 0.001**
Prior surgery^d^	43(25.44)	22(30.14)	21(21.88)	0.222
Vascular interventional	29(17.16)	15(20.55)	14(14.58)	0.308
Use of anticoagulant agent^e^				
Antiplatelet agent	42(24.85)	28(38.36)	14(14.58)	** < 0.001**
Anticoagulant agent^f^	65(38.46)	32(43.84)	33(34.38)	0.211

Values are shown as Mean (SD), Median (IQR), or n (%)

Bold *p* values indicate statistical significance (*p* < 0.05)

*CHD* Coronary heart disease; *AF* indicates atrial fibrillation; *IE* Infective endocarditis; *VD* valvular disease; *SLE* systemic lupus erythematosus; *APS* antiphospholipid syndrome; *ECH* extracranial hemorrhage

Group EGO: Patients with early good outcomes

Group EPO: Patients with early poor outcomes

aOverweight indicates BMI ≥ 24kg/m^2^

^b^It did not include hypertension in our study

^c^It included ECH within 4 weeks prior to intracranial hemorrhage

^d^It included surgeries within 1 month prior to intracranial hemorrhage

^e^It included use within 2 weeks prior to intracranial hemorrhage

^f^Among these patients, 50 remained on anticoagulation within one day of onset. 27 patients experienced an early poor outcome, while 23 did not, with no significant difference between the two groups (28.13% vs. 31.51%, p = 0.633)

### Clinical Characteristics

Among the cohort, 135 patients had intraparenchymal hemorrhage, 27 had pure subarachnoid hemorrhage, 1 had pure IVH, 5 had pure subdural hemorrhage, and 1 had pure epidural hemorrhage. Patients with an early poor outcome had larger hematoma volume (18.17 [IQR 7.57–38.75] mL vs. 5.53 [IQR 0.84–18.12] mL; *p* = 0.002), an increased presence of IVH (21.9% vs. 8.2%; *p* = 0.016), a higher baseline mRS score (5 [IQR 5–5] vs. 4 [IQR 2–5]; *p* < 0.001) and ICH score (2 [IQR 1–3] vs. 1 [IQR 0–1]; *p* < 0.001), and a lower GCS score (4 [IQR 2.5–11] vs. 15 [IQR 11–15]; *p* < 0.001). Furthermore, the percentage of patients with baseline mRS scores ≥ 4 (92.2% vs. 56.2%; *p* < 0.001), GCS scores ≤ 8 (65.6% vs. 16.4%; *p* < 0.001) and ICH scores ≥ 3 (35.4% vs. 8.2%; *p* < 0.001) was significantly higher among patients with an early poor outcome. Only 15 (8.9%) patients received neurosurgical intervention after in-hospital ICH, with no significant difference between the two groups (*p* = 0.776). Seventy (41.4%) patients were admitted to the ICU, with significantly higher ICU occupancy among those with an early poor outcome (54.2% vs. 24.7%; *p* < 0.001). Patients with an early poor outcome had a higher incidence of infectious complications (43.8% vs. 24.7%; *p* = 0.010), particularly pneumonia (33.3% vs. 16.4%; *p* = 0.013). Table 2 summarizes the clinical characteristics of the patients, grouped according to outcome.

**Table 2:** Clinical characteristics and treatments of patients with in-hospital ICH at baseline

	Overall N = 169	Group EGO N = 73	Group EPO N = 96	*P* value
SBP at onset^a^	133.5(123–150.5)	131.5(123.5,150.5)	134(122.5,150)	0.824
DBP at onset^b^	74(65.5–84.5)	74.5(67.5,84.0)	74(64.5,84.5)	0.814
Symptomatic	134(79.29)	54(73.97)	80(83.33)	0.137
Baseline GCS score	10(3–15)	15(11,15)	4(2.5,11)	** < 0.001**
GCS ≤ 8	75(44.37)	12(16.44)	63(65.63)	** < 0.001**
Baseline mRS score	5(4–5)	4(2,5)	5(5,5)	** < 0.001**
mRS ≥ 4	130(76.92)	41(56.16)	89(92.17)	** < 0.001**
Baseline ICH-Score	1(0–2)	1(0,1)	2(1,3)	** < 0.001**
ICH ≥ 3	40(23.67)	6(8.22)	34(35.42)	** < 0.001**
Baseline volume^c^	14.18(2.47,30.21)	5.53(0.84,18.12)	18.17(7.57,38.75)	**0.002**
Location bleeding^d^				
Supratentorial	116(68.64)	49(67.12)	67(69.79)	0.711
Infratentorial	23(13.61)	8(10.96)	15(15.63)	0.381
Intraventricular	27(15.98)	6(8.22)	21(21.88)	**0.016**
Extradural	2(1.18)	2(2.74)	0	0.185
Subdural	8(4.73)	4(5.48)	4(4.17)	0.691
Subarachnoid Hemorrhage	37(21.89)	16(21.92)	21(21.88)	0.995
Lobe	80(47.34)	32(43.84)	48(50.00)	0.427
Laboratory features				
Hemoglobin, g/L	96(81,122)	114(92,129)	86(71.5,102)	** < 0.001**
Anemia	106(62.72)	30(41.10)	76(79.17)	**0.001**
Platelet, 10^9^/L	132(57,233)	167(132,253)	85(24,198.5)	** < 0.001**
Thrombocytopenia	68(40.24)	13(17.81)	55(57.29)	** < 0.001**
WBC, 10^9^/L	8.55(5.57,12.43)	8.31(6.35,11.12)	8.62(4.81,13.70)	0.900
ALT, U/L	23(14,51)	21(12,32)	27(16,68.5)	0.074
SCr, umol/L	74(57,113)	71(53,85)	85(60,135.5)	**0.007**
SCr > 104	48(28.40)	10(13.70)	38(39.58)	** < 0.001**
INR	1.13(1.05,1.31)	1.07(1.01,1.14)	1.23(1.07,1.40)	** < 0.001**
INR ≥ 1.5	22(13.02)	5(6.85)	17(17.71)	**0.038**
APTT, s	31.40(26.10, 39.60)	28.70(25.50, 33.20)	34.95(28.80, 43.45)	** < 0.001**
APTT > 32.5	77(45.56)	21(28.77)	56(58.33)	** < 0.001**
TT, s	17.60(16.40, 20.10)	17.50(16.50, 18.70)	17.65(16.40, 20.45)	0.381
TT > 21.0	30(17.75)	10(13.70)	20(20.83)	0.229
Treatment				
Neurosurgery therapy	15(8.88)	7(9.59)	8(8.33)	0.776
ICU admission	70(41.42)	18(24.66)	52(54.17)	** < 0.001**
Days in ICU	4.5(1,14)	11.5(2,28)	4(1,11.5)	**0.049**
Days from onset to discharge	7(1,18)	18(11,35)	2.0(0,6.5)	** < 0.001**
Complication infection	60(35.50)	18(24.66)	42(43.75)	**0.010**
Pneumonia	44(26.04)	12(16.44)	32(33.33)	**0.013**
Intracranial Infection	9(5.33)	2(2.74)	7(7.29)	0.302
Blood infection	8(4.73)	2(2.74)	6(6.25)	0.468

Values are shown as Median (IQR), or n (%)

Bold *p* values indicate statistical significance (*p* < 0.05)

*SBP* Systolic blood pressure; *DBP* Diastolic blood pressure; *WBC* White blood cell; *ALT* Alanine Aminotransferase; *SCr* Serum creatinine; *INR* International normalized ratio; *APTT* Activated partial thromboplastin time; *TT* Thrombin time; *ICU* Intensive care unit

Group EGO: Patients with early good outcomes

Group EPO: Patients with early poor outcomes

^a^120 patients were included

^b^120 patients were included

^c^Patients with pure intraventricular, subdural, or subarachnoid hemorrhages (volumes not calculated) were excluded

^d^Location include parenchymal bleeds that extend into other anatomical compartments (e.g., intraventricular or subarachnoid spaces)

### Etiology

The etiology of in-hospital ICH is summarized in Table 3. The most frequent causes were systemic disease (39.6%) and medication use (36.7%), followed by structural vascular lesions (10.7%). Less common causes, including undetermined causes (6.5%), hypertension (3.6%), multiple causes (2.4%), and amyloid angiopathy (0.6%), were grouped into a category labeled “other causes” for simplification. Patients with an early poor outcome had a higher prevalence of systemic disease etiology (55.2% vs. 19.2%) but a lower prevalence of medication-related causes (29.2% vs. 46.6%) and structural vascular lesions (6.3% vs. 16.4%) compared to those without an early poor outcome. Notably, among all patients with in-hospital ICH, only 6 (3.6%) cases were attributed to hypertension, and none of them developed an early poor outcome. The specific systemic diseases contributing to ICH are detailed in eTable 1.

**Table 3:** Causes of in-hospital ICH. (modified SMASH-U)

	Overall N = 169	Group EGO N = 73	Group EPO N = 96	*P* value
Etiology				** < 0.001**
Structural vascular lesions	18(10.65)	12(16.44)	6(6.25)	
Medication	62(36.69)	34(46.58)	28(29.17)	
Systemic disease	67(39.64)	14(19.18)	53(55.21)	
Others	22(13.02)	13(17.81)	9(9.38)	
CAA	1(0.59)	1(1.37)	0	
Hypertension	6(3.55)	6(8.22)	0	
Undetermined	11(6.51)	6(8.22)	5(5.21)	
Multiple causes	4(2.37)	0	4(4.17)	

Values are shown as n(%)

Bold *p* values indicate statistical significance (*p* < 0.05)

*CAA* Amyloid angiopathy

Group EGO: Patients with early good outcomes

Group EPO: Patients with early poor outcomes

### Multivariate Regression Analysis

The results of univariate and multivariate logistic regression analyses are presented in Table 4. Univariate analysis showed that age, dyslipidemia, prior ischemic stroke, hematologic tumor, prior ECH, antiplatelet agent use, baseline hematoma volume, presence of IVH, baseline mRS score ≥ 4, baseline GCS score ≤ 8, baseline ICH score ≥ 3, anemia, thrombocytopenia, elevated SCr level, INR ≥ 1.5, ICU admission, pneumonia, and etiology were associated with an early poor outcome (*p* < 0.05). Among the various etiologies analyzed, systemic disease was significantly associated with a higher risk of early poor outcomes. Therefore, in the subsequent multivariable logistic regression analysis, etiology was categorized into two groups: systemic disease and nonsystemic disease. Based on forward stepwise elimination, prior ECH (odds ratio [OR] 4.65, 95% confidence interval [CI] 1.46–14.76, *p* = 0.009), baseline mRS score ≥ 4 (OR 11.17, 95% CI 2.90–43.03, *p* < 0.001), baseline GCS score ≤ 8 (OR 6.63, 95% CI 2.56–17.20, *p* < 0.001), and systemic disease etiology (OR 7.05, 95% CI 2.09–23.79, *p* = 0.002) were identified as independent risk factors for an early poor outcome. No significant collinearity was observed for any of the variables included in the model (eTable 2). Sensitivity analyses incorporating hematoma volume and dyslipidemia showed consistent results, as detailed in eTable 3.

**Table 4:** Univariate and multivariate logistic analysis model of early poor outcome of in-hospital ICH

	Univariate		Multivariate	
	OR (95%CI)	*P* value	OR (95%CI)	*P* value
Age, years	0.97(0.95–0.99)	0.002		
Age ≤ 45	2.43(1.20–4.89)	0.013		
Dyslipidemia	0.22(0.07–0.72)	0.012		
Prior ischemic stroke	0.36(0.19–0.69)	0.002		
Hematologic tumor	4.04(1.45–11.27)	0.008		
Prior ECH	6.02(2.69–13.46)	< 0.001	4.65(1.46–14.76)	0.009
Use of antiplatelet agent	0.27(0.13–0.57)	< 0.001		
Volume, mL	1.02(1.00–1.03)	0.028		
Presence of IVH	3.13(1.19–8.21)	0.021		
Baseline mRS ≥ 4	9.92(4.04–24.35)	< 0.001	11.17(2.90–43.03)	< 0.001
Baseline GCS ≤ 8	9.70(4.59–20.51)	< 0.001	6.63(2.56–17.20)	< 0.001
Baseline ICH score ≥ 3	6.12(2.41–15.58)	< 0.001		
Anemia	3.55(1.60–7.90)	0.002		
Thrombocytopenia	6.19(3.00–12.76)	< 0.001		
SCr > 104umol/L	4.13(1.89–9.03)	< 0.001		
INR ≥ 1.5	2.93(1.03–8.35)	0.048		
APTT > 32.5	3.47(1.81–6.64)	< 0.001		
ICU admission	3.61(1.85–7.03)	< 0.001		
Pneumonia	2.54(1.20–5.38)	0.015		
Etiology^a^		< 0.001		
Structural vascular lesions	0.72(0.20–2.64)	0.077		
Medication	1.19(0.44–3.19)	0.419		
Systemic disease	5.47(1.94–15.38)	< 0.001	7.05(2.09–23.79)	0.002

*ECH* Extracranial hemorrhage; *IVH* Intraventricular hemorrhage; *SCr* Serum creatinine; *INR* International normalized ratio; *APTT* Activated partial thromboplastin time; *ICU* Intensive care unit

^a^Using “other causes” as the reference variable in univariate logistic analysis

### Subgroup Analysis

Subgroup analysis (eTable 4) demonstrated a consistent association of prior ECH, baseline mRS score ≥ 4, baseline GCS score ≤ 8, and systemic disease etiology with early poor outcomes in in-hospital ICH across all subgroups, with no significant interactions observed.

### Nomogram Model

**Fig. 2:**
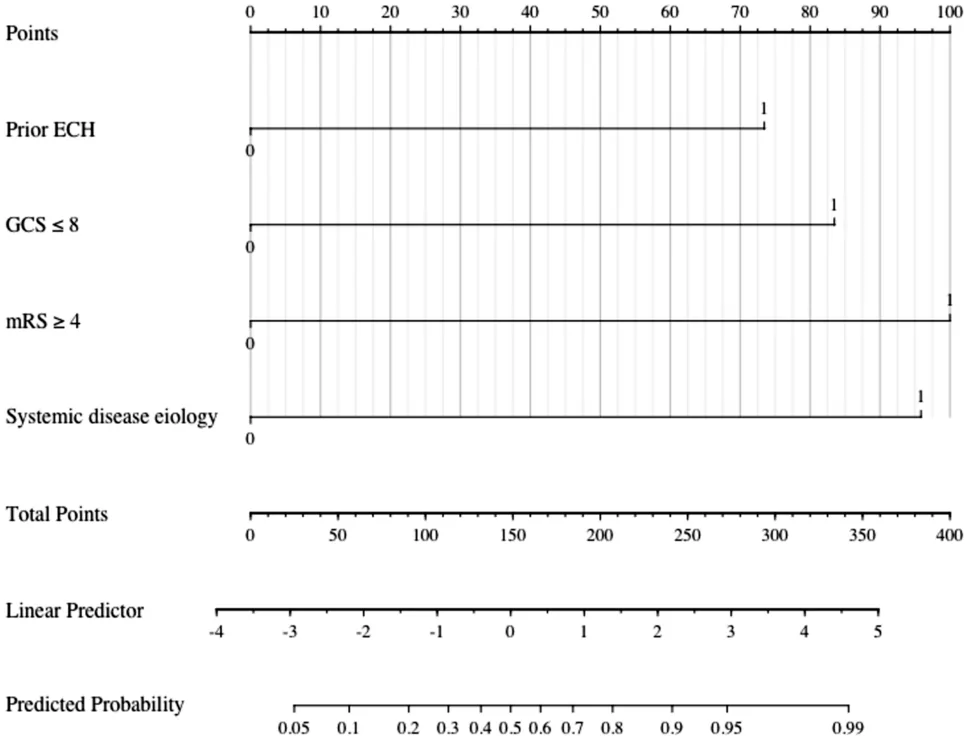
IH‐ICH nomogram for predicting the probability of early poor outcome after in-hospital ICH. ECH extracranial hemorrhage, GCS Glasgow Coma Scale, IH‐ICH in‐hospital intracranial hemorrhage, mRS modified Rankin Scale

**Fig. 3:**
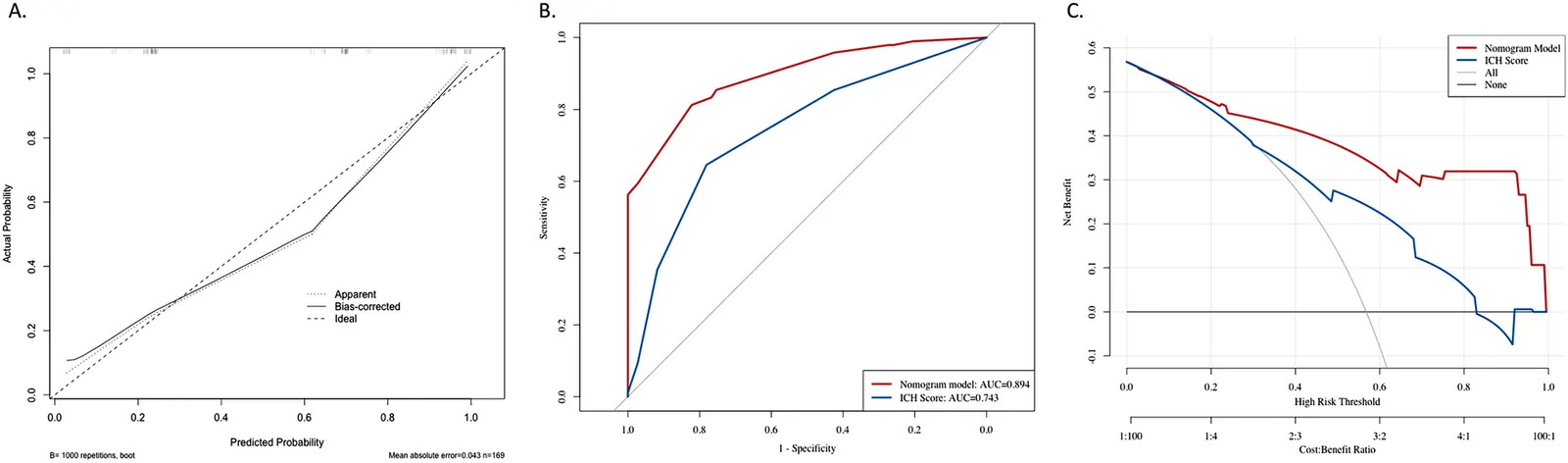
**a** Calibration plot for nomogram‐predicted probability of early poor outcome. **b** Receiver operating characteristic curves detect the discriminative ability of our nomogram and ICH score in the cohort. **c** Decision curve analysis of our nomogram and ICH score in the cohort. AUC area under the curve, ICH intracranial hemorrhage

The IH‐ICH nomogram model was constructed using prior ECH, baseline mRS score ≥ 4, baseline GCS score ≤ 8, and systemic disease etiology (Fig. 2). Calibration plots further confirmed excellent accuracy between the predicted and actual probabilities (mean absolute error = 0.043) (Fig. 3a). Moreover, the AUROC of the nomogram for predicting early poor outcomes was 0.894, which was significantly higher than the AUROC of 0.743 for the ICH score (*p* < 0.001) (Fig. 3b). Subgroup analyses conducted in patients with intracerebral hemorrhage and HT-IS confirmed the robustness of the model, with consistent performance observed across these populations (eFigure 2). Finally, the DCA curve highlighted the clinical value of the model. The IH-ICH nomogram model provided a positive net benefit across threshold probabilities ranging from 10 to 90%, which was clearly better than that of the ICH score (Fig. 3c).

## Discussion

In this study, the in-hospital death/DAMA rate for patients with in-hospital ICH was 56.8%, which is significantly higher than the in-hospital death/DAMA rate for community-onset ICH (21.8%) in China [20]. Among patients with DAMA, 88% had an mRS score of 5 at discharge, and 8% had an mRS score of 4, suggesting that most of these patients were in a near-death state. A study from Europe reported that the in-hospital mortality rate for in-hospital ICH was 57.7% (*n* = 26) [9], which aligns with our findings. The high in-hospital mortality for in-hospital ICH is likely attributable to the critical condition of hospitalized patients and the frequent presence of severe underlying comorbidities [8].

Significant independent risk factors for early poor outcomes included prior ECH, severe coma (GCS score ≤ 8), worse baseline functional status (mRS score ≥ 4), and systemic disease etiology. Spontaneous ECH is often associated with underlying coagulopathies and vascular structural abnormalities, which may increase the risk of ICH [21–24]. In this study, patients with prior ECH had lower platelet counts (43 × 10^9^/Lvs. 165 × 10^9^/L; *p* < 0.001) and higher INR values (1.22 vs. 1.10; *p* = 0.002). Therefore, the association between prior ECH and outcomes may be attributed to underlying coagulation abnormalities, hemostatic alterations, and prolonged bleeding. Patients with prior ECH had larger hematoma volumes at baseline, although this difference was not statistically significant (15.7 vs. 9.4 mL; *p* = 0.147). Further studies are needed to investigate the underlying mechanisms.

In community-onset ICH, hypertension is the most common cause in China [3]. However, in our in-hospital ICH cohort, the most common cause was secondary hemorrhage, including systemic disease (39.6%) and medication (36.7%), with only six cases of hypertension-related ICH. The patients with in-hospital ICH caused by systemic disease had a higher risk of early poor outcomes compared to those with in-hospital ICH caused by nonsystemic disease (OR 7.05, 95% CI 2.09–23.79, *p* = 0.002). This may be because systemic disease etiology was difficult to correct immediately, and the occurrence of ICH further complicated the patient’s condition, making treatment more challenging. Similar to community-onset ICH [25–27], severe coma (GCS score ≤ 8) remains an independent prognostic factor in in-hospital ICH, as well as worse functional status (mRS score ≥ 4) [28]. This is primarily because immediate GCS and mRS scores, as critical indicators of neurological damage and functional status, provide a more accurate assessment of the patient’s overall condition. Consequently, both measures serve as reliable and independent risk factors for poor prognosis in patients with in-hospital ICH, who typically present with more complex underlying conditions. Interestingly, we observed that prior ischemic stroke and prior antiplatelet use were associated with a lower OR for poor outcome in univariate analysis, which appears counterintuitive. This may be partly explained by the unique context of in-hospital ICH. Patients with IH-ICH are typically hospitalized for critical illness and generally have poor premorbid status, potentially diminishing the impact of these prior factors on outcomes. Moreover, patients with prior ischemic stroke or those on antiplatelet therapy may have received more intensive monitoring during hospitalization, facilitating earlier detection and intervention. We further examined whether this association was driven by cases of HT-IS, which are often associated with these risk factors. However, even after excluding HT-IS cases, the associations remained consistent: prior ischemic stroke (OR 0.422, 95% CI 0.20–0.91, *p* = 0.027) and antiplatelet use (OR 0.31, 95% CI 0.13–0.74, *p* = 0.008). Importantly, these associations were no longer statistically significant in multivariable analysis, suggesting the initial findings may reflect confounding by indication or severity rather than a true protective effect. Therefore, these findings should be interpreted with caution and validated in larger cohorts.

We further constructed a novel nomogram model to predict the post‐ICH outcome status by applying these independent risk factors. This model showed great predictive value, with an AUROC of 0.894, which is significantly superior to that of the ICH score (*p* < 0.001). The ICH score, developed by Dr. J. Claude Hemphill and colleagues in 2001, is a clinical grading scale specifically designed to predict the prognosis of patients with intracerebral hemorrhage. The overall score ranges from 0 to 6, with higher scores indicating worse prognosis [29–31]. Although the ICH score may not be fully applicable to nonparenchymal hemorrhages, which were included in our study population, subgroup analyses demonstrated that the nomogram model consistently outperformed the ICH score in the intracerebral hemorrhage subgroup, as well as the HT-IS subgroup, which may have distinct pathophysiological mechanisms. These results highlight the reliability and generalizability of the IH-ICH nomogram across diverse clinical scenarios. The lower predictive value of the ICH score compared to the IH-ICH nomogram in in-hospital ICH might be explained by the different mechanisms underlying in-hospital ICH, as mentioned before. Systemic factors, including severe underlying diseases and comorbidities, often have a greater impact on outcomes than the initial hemorrhagic characteristics, such as hemorrhage volume and location, which are included in the ICH score. As a multivariable prediction tool, the advantage of the nomogram lies in its ability to support clinical decision-making. It is particularly useful in predicting risks in complex cases. Consequently, the nomogram has been widely applied in cancer, cardiovascular diseases, and surgery [32, 33].

To the best of our knowledge, this is the first study that systematically investigated the independent risk factors for early poor outcomes among patients with in-hospital ICH. Our study was strengthened by the inclusion of a large hospital-based sample of consecutive patients and objective electronic medical records, thus eliminating the potential bias from self-report and selective inclusion. In addition, key information such as stroke severity, comorbid conditions, treatment strategies, and outcome measures was documented, with minimal missing data.

A few limitations should be considered. First, our study is retrospective, and due to the low incidence of in-hospital ICH, the sample size is relatively small. Our predictive model lacks external validation and requires confirmation through future multicenter prospective studies. Furthermore, the high proportion of DAMA patients in our study may introduce potential bias related to self-fulfilling prophecies. Although DAMA in China typically reflects terminal status and a preference for home-based end-of-life care, as supported by the high mRS scores at discharge in our cohort, residual bias remains, as postdischarge outcomes were not tracked. Future multicenter prospective studies with postdischarge follow-up are needed to validate our findings. Additionally, tools such as the ICH score, SMASH-U classification, and the hematoma volume estimation method ([a × b × c]/2), while widely used, may not be well-suited for accurately assessing nonparenchymal hemorrhages. To maintain consistency in evaluation, we retained these tools, but readers should interpret the corresponding results with caution.

## Conclusions

In conclusion, our study highlights that prior ECH, severe coma (GCS score ≤ 8), poor baseline functional status (mRS score ≥ 4), and systemic disease etiology are important independent risk factors for early poor outcomes in in-hospital ICH. The IH-ICH nomogram, which integrates these factors, demonstrates superior predictive value compared to the ICH score and would be helpful for early identification and monitoring for high-risk patients. Further validation through multicenter, prospective, high-quality studies is needed.

## Supplementary Information

Below is the link to the electronic supplementary material.

Supplementary file1 (DOCX 2903 KB)

## Data Availability

All data that support the findings of this study are available from the corresponding author upon reasonable request.
