# Supplementary material for: Risk Factors for Early Poor Outcomes in In-hospital Intracranial Hemorrhage: A Retrospective Cohort Study
Source: Neurocrit Care. 2025 Jul 1;43(3):968–77. doi: 10.1007/s12028-025-02306-0 (PMC12647189; doi:10.1007/s12028-025-02306-0)

**Supplementary material**

**eFigure 1. Death causes of in-hospital spontaneous intracerebral hemorrhage.**


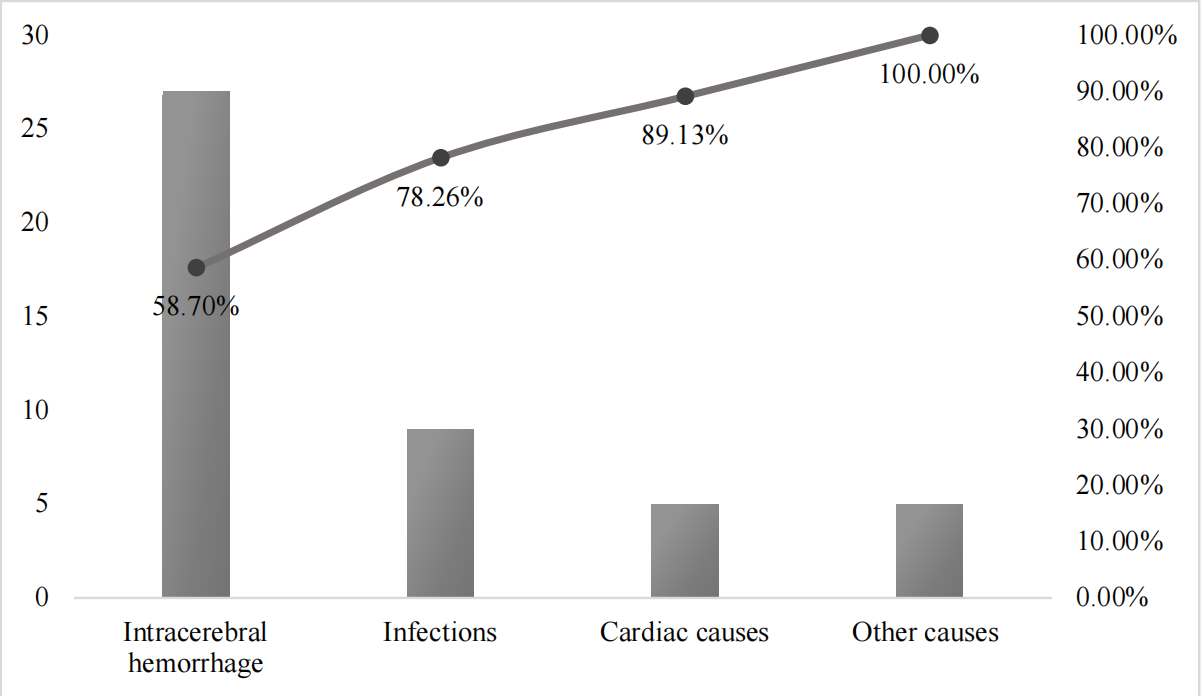


e**Table 1. Systemic Diseases Etiology for In-Hospital ICH**

|  | Overall  N=67 | Group EGO  N=14 | Group EPO  N=53 |
| --- | --- | --- | --- |
| **Hematologic Diseases** | **31(46.27)** | **5(35.71)** | **26(49.06)** |
| Malignancies | 23(34.33) | 4(28.57) | 19(35.85) |
| Thrombocytopenia | 7(10.45) | 1(7.14) | 6(11.32) |
| Others | 1(1.49) | 0 | 1(1.89) |
| **Infectious Diseases** | **17(25.37)** | **5(35.71)** | **12(22.64)** |
| Intracranial Infection | 9(13.43) | 1(7.14) | 8(15.09) |
| Infective Endocarditis | 5(7.46) | 4(28.57) | 1(1.89) |
| Others | 3(4.48) | 0 | 3(5.66) |
| **Autoimmune Diseases** | **12(17.91)** | **2(14.29)** | **10(18.87)** |
| SLE | 7(10.45) | 1(7.14) | 6(11.32) |
| Others | 5(7.46) | 1(7.14) | 4(7.55) |
| **Other Systemic Conditions** | **7(10.45)** | **2(14.29)** | **5(9.43)** |

Values are shown as n(%)

Abbreviations: SLE, systemic lupus erythematosus.

Group EGO: Patients with early good outcomes.

Group EPO: Patients with early poor outcomes.

**eTable 2. Collinearity analysis of variables included in multivariate logistic regression model.**

| Variables | Age≤45 years | Sex | Prior IS | HT | Prior ECH | Use of AT agent | IVH | Etiology | mRS≥4 |
| --- | --- | --- | --- | --- | --- | --- | --- | --- | --- |
| VIF | 1.21 | 1.15 | 1.41 | 1.51 | 1.54 | 1.65 | 1.44 | 1.22 | 1.45 |

| Variables | GCS≤8 | ICH≥3 | Anemia | Thrombocytopenia | SCr>104 | INR≥1.5 | ICU admission | Pneumonia |
| --- | --- | --- | --- | --- | --- | --- | --- | --- |
| VIF | 1.74 | 2.04 | 1.16 | 1.67 | 1.52 | 1.15 | 1.34 | 1.22 |

Abbreviations: IS, ischemic stroke; HT, hematologic tumor; ECH, extracranial hemorrhage; AT, antiplatelet; IVH, intraventricular hemorrhage; ICU, intensive care unit.

**eTable 3.** **Multivariate logistic regression model additionally included baseline hematoma volume and dyslipidemia.**

|  | Model 1 | | Model 2 | | Model 3 | |
| --- | --- | --- | --- | --- | --- | --- |
|  | OR (95%CI) | P value | OR (95%CI) | P value | OR (95%CI) | P value |
| Prior ECH | 4.32(1.33-14.09) | 0.015 | 6.41(1.51-27.15) | 0.012 | 7.63(1.81-32.12) | 0.006 |
| Baseline mRS≥4 | 10.95(2.79-42.92) | <0.001 | 8.79(1.91-40.44) | 0.005 | 8.22(1.73-39.08) | 0.008 |
| Baseline GCS≤8 | 6.74(2.53-18.00) | <0.001 | 4.35(1.35-14.09) | 0.014 | 4.03(1.23-13.14) | 0.021 |
| Systemic disease cause | 6.51(1.90-22.25) | 0.005 | 10.50(2.45-44.85) | 0.002 | 10.57(2.43-46.00) | 0.002 |

Abbreviations: ECH, extracranial hemorrhage.

Model 1: Dyslipidemia included;

Model 2: Baseline hematoma volume included;

Model 3: Baseline hematoma volume and dyslipidemia included.

**eTable 4. Interaction analysis of prior ECH, baseline mRS≥4, baseline GCS≤8 and systematic diseases etiology.**

|  | Prior ECH | | Baseline mRS≥4 | |
| --- | --- | --- | --- | --- |
|  | OR (95%CI) | P value for interaction | OR (95%CI) | P value for interaction |
| Age |  | 0.792 |  | 0.961 |
| ≤45 years | 4.71(1.14-19.48) |  | 9.92(2.09-47.02) |  |
| >45 years | 5.94(2.20-16.03) |  | 10.41(3.33-32.56) |  |
| Sex |  | 0.305 |  | 0.712 |
| Female | 11.76(2.54-54.49) |  | 11.79(3.04-45.72) |  |
| Male | 4.51(1.65-12.30) |  | 8.39(2.52-27.87) |  |
| Baseline volume, mL | - | 0.826 | - | 0.676 |
| Presence of IVH |  | 0.828 |  | 0.867 |
| Yes | 8.18(0.34-195.16) |  | 11.78(0.12->999.99) |  |
| No | 5.69(2.49-13.03) |  | 7.88(3.20-19.43) |  |
| Thrombocytopenia |  | 0.496 |  | 0.383 |
| Yes | 5.40(1.33-21.89) |  | 20.40(4.50-92.47) |  |
| No | 2.90(0.96-8.76) |  | 8.44(2.34-30.49) |  |
| ICU admission |  | 0.357 |  | 0.621 |
| Yes | 3.67(0.95-14.23) |  | 15.92(0.37-684.46) |  |
| No | 8.17(2.91-22.95) |  | 5.98(2.30-15.55) |  |
| Pneumonia |  | 0.852 |  | 0.711 |
| Yes | 5.00(0.94-26.53) |  | 15.49(0.35-682.37) |  |
| No | 6.00(2.37-15.20) |  | 7.42(2.96-18.63) |  |

|  | Baseline GCS≤8 | | Systemic disease etiology | |
| --- | --- | --- | --- | --- |
|  | OR (95%CI) | P value for interaction | OR (95%CI) | P value for interaction |
| Age |  | 0.793 |  | 0.906 |
| ≤45 years | 9.36(3.93-22.31) |  | 4.52(1.20-16.96) |  |
| >45 years | 12.00(2.34-61.52) |  | 4.97(2.12-11.63) |  |
| Sex |  | 0.367 |  | 0.371 |
| Female | 6.97(2.45-19.89) |  | 8.16(2.50-26.58) |  |
| Male | 13.95(4.73-41.11) |  | 4.11(1.62-10.39) |  |
| Baseline volume, mL |  | 0.355 |  | 0.133 |
| Presence of IVH |  | 0.259 |  | 0.330 |
| Yes | 3.00(0.37-24.29) |  | 20.64(0.92-464.63) |  |
| No | 11.06(4.63-26.42) |  | 4.21(2.01-8.83) |  |
| Thrombocytopenia |  | 0.323 |  | 0.311 |
| Yes | 24.66(2.97-204.67) |  | 1.92(0.56-6.56) |  |
| No | 7.72(3.10-19.22) |  | 4.37(1.59-12.02) |  |
| ICU admission |  | 0.537 |  | 0.174 |
| Yes | 6.00(1.87-19.21) |  | 37.03(2.01-682.83) |  |
| No | 9.80(3.48-27.58) |  | 4.50(1.91-10.58) |  |
| Pneumonia |  | 0.844 |  | 0.936 |
| Yes | 11.00(2.03-59.74) |  | 5.67(1.07-30.08) |  |
| No | 9.09(3.89-21.24) |  | 5.25(2.36-11.70) |  |

Abbreviations: ECH, extracranial hemorrhage; IVH, intraventricular hemorrhage; ICU, intensive care unit.

**eFigure 2. ROC curves of IH-ICH nomogram and ICH Score in intracerebral hemorrhage patients (A). ROC curves of IH-ICH nomogram and ICH Score in hemorrhagic transformation of ischemic stroke patients (B).**


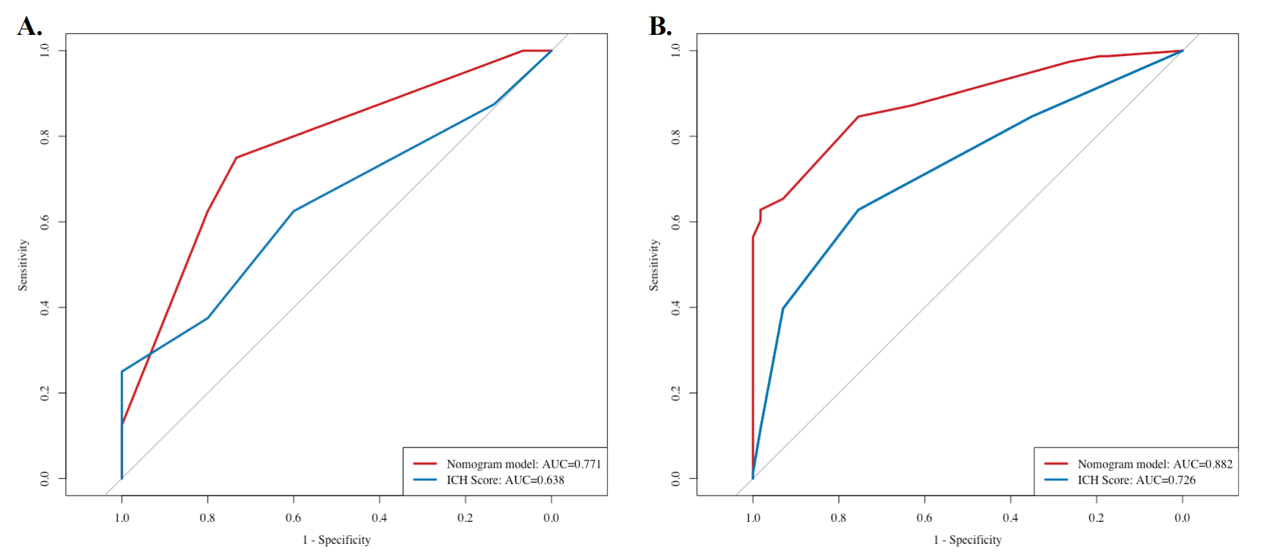

Supplement: Supplementary file 1 — Supplementary file1 (DOCX 2903 KB) [file 12028_2025_2306_MOESM1_ESM.docx]
